# Physical Condition Does Not Affect Gravity-Induced Loss of Consciousness during Human Centrifuge Training in Well-Experienced Young Aviators

**DOI:** 10.1371/journal.pone.0147921

**Published:** 2016-01-26

**Authors:** Jinhee Park, Chul Yun, Seungcheol Kang

**Affiliations:** 1 Seoul National University Hospital, 101 Daehak-ro Jongno-gu, Seoul, 110–744, Republic of Korea; 2 Aerospace Medical Center, Republic of Korea Air Force, P.O. Box 18, Song-am-ri, Namil-myeon, Sangdang-gu, Cheongju-si, Chungcheongbuk-do, Republic of Korea; 3 Department of Orthopaedic Surgery, Asan Medical Center Children’s Hospital, University of Ulsan College of Medicine, 88, Olympic-ro, 43-gil, Songpa-gu, Seoul, 05505, Republic of Korea; Northwestern University Feinberg School of Medicine, UNITED STATES

## Abstract

**Background:**

Consensus on whether physical condition affects the risk of gravity-induced loss of consciousness (G-LOC) has not been reached, and most previous studies about the issue did not include well-experienced aviators. We compared the physical conditions of well-experienced young aviators according to the occurrence of G-LOC during human centrifuge training.

**Methods:**

Among 361 young male aviators on active flight duty with experience in high performance aircrafts for at least 2 years, 350 had full data available and were reviewed in this study. We divided the aviators into the G-LOC group and the non-G-LOC group according to their human centrifuge training results. We then compared their basic characteristics, body composition, physical fitness level, and pulmonary function.

**Results:**

Twenty nine aviators (8.3%) who experienced G-LOC during human centrifuge training in their first trials were classified into the G-LOC group. There was no difference in physical condition of aviators between the two groups.

**Conclusions:**

Young aviators with experience in G-LOC showed no difference in physical condition such as muscle mass, strength, and general endurance from the aviators with no such experience. Although more studies are needed, physical condition does not seem to be a significant determinant of G-LOC among the experienced aviators.

## Introduction

With increasing importance of flight missions to military operations, more high performance aircrafts have been introduced to the military force. Aviators of high performance aircrafts are frequently exposed to high gravitational force (G-force) that results from quick turns or movements. High G-force can cause cerebral hypoxia and even loss of consciousness in humans by disturbing blood supply to the brain [1–3]. G-force–induced loss of consciousness is simply referred to as “G-LOC.” Aircraft accidents due to G-LOC occurred in the First World War [1]but have only been formally reported and studied since the 1980s [4]. When an aviator experiences G-LOC, he or she may experience a lack of purposeful movement for at least 30 seconds even when the high G-force is removed immediately [5]. Therefore, even short-term G-LOC can cause fatal aircraft accidents. To address those concerns, there have been many studies on G-LOC, and much effort has been made to prevent it. Only the G-force applied on the z-axis (head-to-foot direction) is counted and referred to as “G-force” throughout this paper.

An individual’s tolerance to G-force (G-tolerance) depends on factors such as the degree of G-force and the duration of exposure. G-tolerance varies among individuals and can be improved by measures such as anti-G suits, anti-G straining maneuvers (AGSM), positive pressure breathing systems, and reclined seats [6,7]. Among them, AGSM is known to be the most effective method [8,9]. AGSM is broadly divided into 2 stages: the first stage is muscle tensing, and the second stage is the L-1 maneuver. Muscle tensing involves the muscles of the abdomen, buttocks, and extremities. The L-1 maneuver consists of a Valsalva maneuver with a totally closed glottis as well as breathing at intervals of 2.5–3 s [10,11]. Many studies have reported the association between G-tolerance and physical conditioning such as weight training to enhance the effect of AGSM [12–14] and the association between G-tolerance and respiration [15–17]. However, most of them did not find any effect on physical conditioning on G-tolerance or any analytical errors in earlier studies that had assumed the effect of physical conditioning on G-tolerance [3,18,19].

Because aviators are now exposed to increasing maximum acceleration levels with developing high performance aircrafts, the importance of enhancing G-tolerance is being stressed again [20,21]. However, controversy remains on whether physical condition affects G-tolerance [19]. Furthermore, only few studies have targeted well-experienced aviators on active flight duty.

The purpose of this study is to evaluate the association between G-LOC and physical condition parameters among young and well-experienced aviators. Targeting only young and well-experienced aviators who are on their active flight duties in high performance aircrafts, we investigated their basic parameters of physical conditions such as body composition, physical fitness, and pulmonary function and then compared those parameters of the aviators who underwent G-LOC during human centrifuge trainings with those who did not.

## Materials and Methods

This is a retrospective study. This study was ethically approved by the Aerospace Medical Center–Republic of Korea Air Force Institutional Review Board (ASMC-ROKAF IRB).The first ethical approval was obtained on April 4, 2013, and the data collection began on the next day, April 5, 2013. All the data gathered were originally intended for routine medical checkups and education program, not for the study. No further data were gathered or requested for this study. We collected participant data from patient files and medical records retrospectively. The informed consent was exempted from IRB because the following conditions were met: (1) it is practically impossible to obtain the consent from the subjects in the study process, (2) there is no ground to assume the refusal of consent, and (3) extremely low risks may be exposed to subjects even if the agreement is exempted.

### Subject selection

Military aviators in the Republic of Korea Air Force (ROKAF) visit our institute every 1–2 years to undergo routine medical checkups and participate in an aerospace education program as a part of human centrifuge training in order to qualify as military pilots. Regular human centrifuge procedures are a mandatory part of military training to maintain the pilot’s license. Data on male ROKAF aviators (age ≤40 years) on active flight duty who visited our institution between January 2010 and December 2011 were collected. The aircraft type was limited to those exposed to high G-forces, such as fighters, trainers, and attackers (F-4, F-5, F-15K, KF-16, KA-1, KT-1, and T-50). Aviators with <2 years of flight experience with their current aircraft were excluded from the study. Three hundred and sixty-one aviators met the inclusion criteria. Among them, 350 aviators with full available data were investigated.

### Equipment

All human centrifuge training was conducted in a 6.1-m (20 ft) radius centrifuge (G-LAB, ETC, Southampton, PA, USA) at the Aerospace Medical Center, ROKAF. The centrifuge was configured with an upright (13° seat back angle) seat. All subjects were studied during +Gz exposures of rapid onset (6G/s) commencing from +1.2G (idle level) to +9G, which was maintained for 15 seconds, and terminated with a decelerating rate of -3 G/s reaching 1 G. Pneumatic anti-G suit was applied and the AGSM was performed. Positive pressure breathing systems were not used. During the process, head-up display (HUD) videotapes were recorded and monitored by a specialist in training.

### Subject grouping

We divided the aviators into the G-LOC group and the non-G-LOC group according to their results of human centrifuge training during their aerospace education program. When the subjects released the stick or the specialist identified G-LOC on the HUD, the subjects were classified into the G-LOC group. If subjects showed myoclonic or seizure-like activity, involuntary discontinuity of AGSM, or similar signs, the centrifuge was stopped and the subjects were classified into the G-LOC group. A simple loss of peripheral vision without any other symptoms was not regarded as G-LOC. Those subjects who did not experience G-LOC were classified into the non-G-LOC group. Only G-LOC occurring in the first human centrifuge trial during the education program was counted, and the results of any re-trials to pass the aerospace education program were not counted.

### Investigated variables

We compared the subjects’ basic characteristics, body composition, physical fitness level (cardiopulmonary endurance, muscle power, and body flexibility), and results of the pulmonary function test (PFT). All of these were measured in one day, but not on the same day as the centrifuge training. The body composition test could be affected by dehydration due to the physical fitness test, while the cardiopulmonary endurance test could be affected by the increased heartbeat due to the strength test. Therefore, body composition was measured first, followed by the PFT, general endurance, muscle power, and body flexibility.

#### Basic characteristics and body composition

The bioimpedance spectroscopy (BIS) technique (InBody 720; Biospace Co., Ltd., Seoul, Korea) was used to assess the body composition. The test was performed after fasting for a minimum of 4 hours, no exercise for a minimum of 12 hours, abstinence from alcohol for a minimum of 48 hours, and abstinence from diuretics for a minimum of 7 days to minimize the bias. Finally, the subjects voided 30 minutes before the test. Each subject’s height was measured first while they stood on the electrodes embedded in the scale platform of the analyzers wearing only a light gown. The subject then grasped the handles and ensured that all of their fingers contacted the finger electrodes. During the measurement, the subjects kept their limbs slightly abducted and were not allowed to talk.

After these procedures, each subject’s height, age, and gender were recorded, and the following body compositions were analyzed: body mass index (BMI), basic metabolic rate (BMR), percent body fat, waist-hip ratio, skeletal muscle mass (SMM), muscle mass of the upper extremities, muscle mass of the lower extremities, and muscle mass of the trunk.

#### Physical fitness

The InBody u-Town (Biospace Co., Ltd.) was used for the physical fitness assessment. The measured variables were as follows: grip strength test, back strength test, leg strength test (knee extension strength in a 60° flexed position), sit-and-reach test, cardiopulmonary endurance (maximum oxygen consumption), whole body reaction time, sargent jump, number of sit-ups in 30 seconds, and standing on 1 leg with eyes closed.

The grip strength test was performed for 3 seconds with the subject’s mid-phalanx of 2–5 fingers facing the handle. The better of the 2 results was chosen for each hand. Maximum oxygen consumption was measured for cardiopulmonary endurance. Each subject was seated on a bicycle with his knees in a 10° flexed position when fully extended. After the resting heartbeat was checked for 1 minute, the subject pedaled at 50–60 revolutions per minute (RPM) for 6 minutes, and their heart rate was checked again. In the back strength test, the subject stood with their knees extended, feet approximately 15 cm apart, and trunk leaning approximately 30° forward. The subject pulled the handle with his back strength. The test was performed twice, and the better result was chosen. In the whole body reaction test, the subject stood on an electrical mat with his feet apart about his shoulder width and his knees slightly flexed. The subject then removed his feet from the mat immediately after the auditory signal rang. The time gap between the auditory signal and the foot removal was then measured. The test was performed 5 times, and the mean value was chosen. The standing position on one leg with eyes closed was used for the balancing test. The subject stood on one foot on an electrical mat with his hands on his waist. The duration until the other foot contacted the mat or the standing foot moved was measured. The test was performed on both legs, and the mean value was chosen. The sargent jump was used for the quickness test. The subject was not allowed to flex his knees after jumping. The duration from jumping to landing was measured twice, and the better result was chosen. Muscle strength or mass tends to increase according to weight and height. Hence, we additionally divided muscle strength and muscle mass by each subject’s weight and height, and included these values in the analysis.

#### Pulmonary function test

In the PFT, we measured forced vital capacity (FVC), forced expired volume in 1 s (FEV_1_), forced expired volume in half a second (FEV_0.5_), and peak expiratory flow. We also calculated FEV_1_ divided by FVC (FEV_1_/FVC) and FEV_0.5_ divided by FVC (FEV_0.5_/FVC) and included those values in the analysis.

The demographics of the aviators in our study are shown as Table 1.

**Table 1:** Demographics of aviators.

Characteristics		Total (N = 350)
Basic characteristics		
	Age (years)	30.7±4.6
	Height (cm)	175.1±5.3
	Body weight (kg)	74.2±7.8
	Body mass index (kg/m^2^)	24.2±2.2
	Flight year (year)	6.8±4.3
Body composition		
	Basic metabolic rate (kcal/day)	1650.6±121.3
	Percent body fat (%)	19.7±4.8
	Waist-hip ratio	0.86±0.03
	Skeletal muscle mass (kg)	33.6±3.4
	Muscle mass of right arm (kg)	3.3±0.4
	Muscle mass of left arm (kg)	3.2±0.4
	Muscle mass of right leg (kg)	9.4±1.0
	Muscle mass of left leg (kg)	9.3±1.0
	Muscle mass of trunk (kg)	26.0±2.4
Physical fitness		
	Grip strength, left (kg)	41.3±5.2
	Grip strength, right (kg)	43.5±5.0
	Back strength (kg)	110.6±17.4
	Left knee extensor strength (kg)	57.8±16.6
	Right knee extensor strength (kg)	59.2±16.2
	Sit and reach (cm)	7.1±8.2
	whole body reaction time (sec)	0.36±0.05
	Maximum oxygen consumption (ml/kg/min)	49.8±9.9
	Sargent jump (cm)	40.8±5.6
	Sit-up (times/30sec)	27.4±4.5
	Standing on one leg with eyes closed (sec)	43.4±42.7
Pulmonary function test		
	FVC (L)	4.9±0.7
	FEV0.5 (L)	3.2±0.4
	FEV0.5/FVC (%)^a^	65.8±7.2
	FEV1 (L)	4.2±0.6
	FEV1/FVC (%)^b^	86.8±5.8
	Peak expiratory flow (L/sec)	9.5±1.7

^a^Calculated from FVC and FEV0.5

^b^Calculated from FVC and FEV1

### Statistical analysis

The significances of the differences between means were determined using the independent *t*-test. The significances of the differences between frequencies were determined using the chi-square test, while Fisher’s exact test was used in cases in which the frequency number was <5. Associations between subject variables and occurrence of G-LOC were analyzed by the binary logistic regression analysis. To eliminate confounders among the variables, the variables with p values of <0.05 by univariate analysis were included in the multivariate analysis. Significance was accepted for p values < 0.05. Post-hoc power analysis showed that a sample size of 321 and 29 subjects in the respective groups would provide 98.4% power to detect a difference in means assuming an effect size of 0.80 and a 2-sided α of 0.05. Power analysis for the chi-square test showed that a total sample size of 350 would provide 99.9% power to detect a difference assuming an effect size of 0.30, α of 0.05, and degree of freedom of one.

## Results

Of the 350 aviators, 29 (8.3%) experienced G-LOC during their first human centrifuge training trial. There was no difference in physical conditions between G-LOC and non-G-LOC groups (Table 2).The binary logistic regression analysis also did not show any significant association between the physical conditions and the occurrence of G-LOC (Table 3). Therefore, multivariate analysis was not performed. Muscle strength and mass, which were divided by the subject’s weight and height, also showed no statistically significant difference between the groups.

**Table 2:** Differences between G-LOC group and non-G-LOC group.

Characteristics		G-LOC group (n = 29)	Non-G-LOC group (n = 321)	P-value
Basic characteristics				
	Age (years)	30.9±4.6	30.7±4.6	0.759
	Height (cm)	175.6±5.4	175.0±5.3	0.572
	Body weight (kg)	74.1±8.1	74.3±7.8	0.904
	Body mass index (kg/m^2^)	24.0±2.0	24.2±2.2	0.554
	Flight year (year)	6.5±4.4	6.9±4.3	0.690
Body composition				
	Basic metabolic rate (kcal/day)	1659.8±130.9	1649.7±120.6	0.669
	Percent body fat (%)	19.0±4.6	19.8±4.9	0.433
	Waist-hip ratio	0.86±0.03	0.86±0.03	0.406
	Skeletal muscle mass (kg)	33.9±3.6	33.6±3.4	0.675
	Muscle mass of right arm (kg)	3.3±0.4	3.3±0.4	0.799
	Muscle mass of left arm (kg)	3.2±0.4	3.2±0.4	0.967
	Muscle mass of right leg (kg)	9.5±1.1	9.3±1.0	0.470
	Muscle mass of left leg (kg)	9.4±1.1	9.3±1.0	0.530
	Muscle mass of trunk (kg)	26.1±2.6	26.0±2.3	0.826
Physical fitness				
	Grip strength, left (kg)	41.9±5.0	41.3±5.2	0.535
	Grip strength, right (kg)	44.7±4.8	43.4±5.1	0.192
	Back strength (kg)	114.3±15.3	110.3±17.5	0.238
	Left knee extensor strength (kg)	60.7±18.5	57.5±16.4	0.322
	Right knee extensor strength (kg)	61.7±19.9	58.9±15.8	0.389
	Sit and reach (cm)	8.7±8.3	6.9±8.2	0.254
	whole body reaction time (sec)	0.37±0.05	0.36±0.05	0.439
	Maximum oxygen consumption (ml/kg/min)	52.3±9.3	49.5±9.9	0.151
	Sargent jump (cm)	40.8±6.5	40.8±5.5	0.985
	Sit-up (times/30sec)	36.8±3.0	27.4±4.6	0.497
	Standing on one leg with eyes closed (sec)	46.0±43.5	43.2±42.7	0.740
Pulmonary function test				
	FVC (L)	5.0±0.6	4.9±0.7	0.258
	FEV0.5 (L)	3.3±0.5	3.2±0.4	0.083
	FEV0.5/FVC (%)^a^	66.6±8.4	65.7±7.0	0.519
	FEV1 (L)	4.4±0.5	4.2±0.6	0.115
	FEV1/FVC (%)^b^	84.4±6.5	86.8±5.8	0.576
	Peak expiratory flow (L/sec)	9.9±2.0	9.5±1.6	0.188

^a^ Calculated from FVC and FEV0.5

^b^ Calculated from FVC and FEV1

**Table 3:** Results of binary logistic regression for variables associated with the G-LOC.

Characteristics		B(slope)^a^	p-value	OR (95% CI)
Basic characteristics				
	Age (years)	0.013	0.758	1.013 (0.933–1.100)
	Height (Ht) (cm)	0.021	0.571	1.021 (0.950–1.097)
	Body weight (BW) (kg)	-0.003	0.904	0.997 (0.950–1.047
	Body mass index (kg/m^2^)	-0.054	0.553	0.947 (0.791–1.134)
	Flight year (year)	-0.019	0.689	0.982 (0.897–1.075)
Body composition				
	Basic metabolic rate (kcal/day)	0.001	0.668	1.001 (0.998–1.004)
	Percent body fat (%)	-0.032	0.432	0.968 (0.893–1.050)
	Waist-hip ratio	-6.172	0.405	0.002 (0.000–4188.184)
	Skeletal muscle mass (kg)	0.024	0.674	1.024 (0.916–1.146)
	Skeletal muscle mass (kg)/BW	5.323	0.432	205.026 (0.000–119201148.8)
	Skeletal muscle mass (kg)/Ht	0.034	0.784	1.034 (0.813–1.316)
	Muscle mass of right arm (kg)	0.123	0.798	1.130 (0.441–2.898)
	Muscle mass of left arm (kg)	0.020	0.967	1.020 (0.391–2.660)
	Muscle mass of both arms (kg)	0.037	0.881	1.037 (0.643–1.673)
	Muscle mass of both arms (kg)/BW	9.477	0.733	13057.802 (0.000–5.504E+27)
	Muscle mass of both arms (kg)/Ht	-0.015	0.975	0.985 (0.377–2.573)
	Muscle mass of right leg (kg)	0.145	0.468	1.156 (0.781–1.710)
	Muscle mass of left leg (kg)	0.127	0.529	1.136 (0.764–1.687)
	Muscle mass of both legs (kg)	0.068	0.497	1.071 (0.879–1.304)
	Muscle mass of both legs (kg)/BW	9.753	0.337	17200.273 (0.000–7.483E+12)
	Muscle mass of both legs (kg)/Ht	0.148	0.524	1.159 (0.736–1.827)
	Muscle mass of trunk (kg)	0.018	0.825	1.018 (0.866–1.198)
	Muscle mass of trunk (kg)	4.121	0.635	61.647 (0.000–1479512135)
	Muscle mass of trunk (kg)	-0.001	0.995	0.999 (0.705–1.416)
Physical fitness				
	Grip strength, left (kg)	0.023	0.534	1.023 (0.952–1.100)
	Grip strength, right (kg)	0.050	0.193	1.051 (0.975–1.132)
	Grip strength, both (kg)	0.020	0.307	1.021 (0.981–1.061)
	Grip strength, both (kg)/BW	1.527	0.269	4.605 (0.306-69-232)
	Grip strength, both (kg)/Ht	0.035	0.360	1.035 (0.961–1.115)
	Back strength (kg)	0.014	0.238	1.014 (0.991–1.037)
	Back strength (kg)/BW	1.108	0.163	3.029 (0.639–14.356)
	Back strength (kg)/Ht	0.023	0.276	1.023 (0.982–1.066)
	Knee extensor strength, left (kg)	0.011	0.323	1.011 (0.989–1.033)
	Knee extensor strength, right (kg)	0.010	0.389	1.010 (0.988–1.032)
	Knee extensor strength, both (kg)	0.005	0.342	1.005 (0.994–1.017)
	Knee extensor strength, both (kg)/BW	0.451	0.274	1.569 (0.700–3.521)
	Knee extensor strength, both (kg)/Ht	0.009	0.372	1.009 (0.989–1.029)
	Sit and reach (cm)	0.028	0.254	1.028 (0.980–1.079)
	whole body reaction time (sec)	2.969	0.439	19.470 (0.011–35593.580)
	Maximum oxygen consumption (ml/kg/min)	0.032	0.155	1.033 (0.988–1.079)
	Sargent jump (cm)	-0.001	0.985	0.999 (0.934–1.070)
	Sit-up (times/30sec)	-0.029	0.496	0.971 (0.893–1.056)
	Standing on one leg with eyes closed (sec)	0.001	0.739	1.001 (0.993–1.010)
Pulmonary function test				
	FVC (L)	0.314	0.257	1.369 (0.796–2.354)
	FEV0.5 (L)	0.792	0.083	2.208 (0.901–5.412)
	FEV0.5/FVC (%)^b^	1.799	0.517	6.046 (0.026–1405.618)
	FEV1 (L)	0.570	0.114	1.769 (0.872–3.589)
	FEV1/FVC (%)^c^	1.881	0.575	6.563 (0.009–4732.331)
	Peak expiratory flow (L/sec)	0.156	0.189	1.169 (0.926–1.476)

^a^ B is a positive value if the increase of the characteristics is more positively related to G-LOC.

^b^ Calculated from FVC and FEV0.5

^c^ Calculated from FVC and FEV1

## Discussion

We examined the association between G-LOC and physical condition parameters such as physical fitness level, body composition, and pulmonary function by investigating a relatively large number of young and well-experienced aviators during their active flight duties in high performance aircrafts. However, there was no difference in physical conditions between the G-LOC and non-G-LOC groups (Tables 2 and 3). This raises the question of Type II errors given we found no differences in the groups, but the power analysis showed this study had over 90% power.

The InBody 720 is a well-known method of evaluating body composition [22–24]. We used body composition to conduct a more precise and objective investigation on the association between G-LOC and physical condition. To our knowledge, this is the first such study conducted. The body composition of the G-LOC and non-G-LOC groups was not significantly different in our study. Aerobic exercise was traditionally believed to reduce G-tolerance, whereas anaerobic exercise was believed to increase G-tolerance [19]. Some authors have reported that improved G-tolerance after physical conditioning is not due to muscle hypertrophy but rather to physiological alterations such as neuromuscular adaptation [12]. In our study, we examined muscle mass, strength, and general endurance and could not find any difference between the G-LOC and non-G-LOC groups.

The investigated variables in our study included muscle mass and physical fitness as well as PFT for indirectly measuring the intrathoracic pressure during AGSM. Some studies have assumed the importance of lung volume and peak expiratory pressure in the determination of intrathoracic pressure during the Valsalva maneuver [25–27]. Using the PFT, we measured FVC, FEV_1_, FEV_1_/FVC, FEV_0.5_, FEV_0.5_/FVC, and peak expiratory pressure, but we found no statistically significant difference between the 2 groups. On the other hand, Bain et al. [15] suggested that respiratory muscle fatigue is responsible for G-tolerance. This cannot be determined by our study findings because we did not measure this parameter.

This study investigated only the basic parameters of physical condition. The authors believe that the group action of the muscles or coordination is very important for G-tolerance. Although the data are not shown, almost all the aviators eventually completed the human centrifuge program at their second or third attempts. Although they eventually passed their tests, the authors do not think that their physical condition has been improved within such a short period. We think the improvement of G-tolerance originates from the improvement of coordination and adaptation to effective timing during AGSM. This is also the reason why we divided the subjects into the G-LOC and non-G-LOC groups according to only the results of the first trial. Future studies on the coordination and adaptation problem during AGSM surely are needed. The daily condition of the aviator and/or unexpectedly abrupt G-forces during flight are more likely to be responsible for very rare G-LOC related flight accidents, especially in highly-experienced pilots.

Our findings should be interpreted in the context of some considerations. First, many previous studies investigated whether G-tolerance could be increased by several weeks of weight training. However, a large study was nearly impossible, and the bias caused by the degree of weight training or learning effect from the experience of G-force was a disadvantage [18,19]. In this aspect, our study might have reduced bias by investigating a relatively large number of well-experienced aviators who had flown high performance aircrafts for >2 years after their initial flight training. Second, although the applied G-force was very high (9G), the duration was only 15 seconds. Therefore, it is difficult to determine the effect of muscle fatigue on G-tolerance in our study. Furthermore, because our study was performed using a human centrifuge test and not an actual flight, the assessment of G-LOC due to unexpected G-forces that could happen during an actual flight was also difficult. Third, we assessed the differences in physical condition in a cross-sectional study; therefore, it cannot be concluded that physical conditioning such as weight training is irrelevant for increasing G-tolerance in each individual. Fourth, our study did not include variables such as the daily condition of the aviators, nor did it include flight hours or G-force exposure in real flight environments, which might more accurately reflect each subject’s flight experience. Likewise, we only investigated the basic parameters for physical condition. The action of muscle group, muscle tensing, or coordination was not investigated. For example, although we included PTF as a substitute for Valsalva maneuver, effective timing and coordination with other muscle tensing seem to be more important than expiratory force alone. The present study also did not include other possible functional causes of G-LOC such as jugular vein geometry [28]. Fifth, the physical conditions were compared only among military aviators. The physical condition might affect the G-tolerance because military aviators are already physically conditioned subjects compared to the general population. However, authors believe this study is still meaningful because it is extremely rare for the general population to be exposed to such high G force during flight.

## Conclusions

Physical conditions including muscle mass, strength, and general endurance were not associated with G-LOC in well-experienced aviators. Although more studies are needed, physical condition does not seem to be a significant determinant of G-LOC among the experienced aviators.
